# Evaluating the contribution of plant metabolic pathways in the light to the ATP:NADPH demand using a meta-analysis of isotopically non-stationary metabolic flux analyses

**DOI:** 10.1007/s11120-024-01106-5

**Published:** 2024-06-14

**Authors:** Kaila Smith, Deserah D. Strand, Berkley J. Walker

**Affiliations:** 1 Michigan State Unversity - Department of Energy, Plant Research Laboratory, Michigan State University, East Lansing, MI 48824 USA; 2 Department of Plant Biology, Michigan State University, East Lansing, MI 48824 USA; 3 Plant Biotechnology for Health and Sustainability Program, Michigan State University, East Lansing, MI 48824 USA

**Keywords:** Photorespiration, Energy Demand, Starch synthesis, Rubisco, Central metabolism

## Abstract

Balancing the ATP: NADPH demand from plant metabolism with supply from photosynthesis is essential for preventing photodamage and operating efficiently, so understanding its drivers is important for integrating metabolism with the light reactions of photosynthesis and for bioengineering efforts that may radically change this demand. It is often assumed that the C3 cycle and photorespiration consume the largest amount of ATP and reductant in illuminated leaves and as a result mostly determine the ATP: NADPH demand. However, the quantitative extent to which other energy consuming metabolic processes contribute in large ways to overall ATP: NADPH demand remains unknown. Here, we used the metabolic flux networks of numerous recently published isotopically non-stationary metabolic flux analyses (INST-MFA) to evaluate flux through the C3 cycle, photorespiration, the oxidative pentose phosphate pathway, the tricarboxylic acid cycle, and starch/sucrose synthesis and characterize broad trends in the demand of energy across different pathways and compartments as well as in the overall ATP:NADPH demand. These data sets include a variety of species including *Arabidopsis thaliana*, *Nicotiana tabacum*, and *Camelina sativa* as well as varying environmental factors including high/low light, day length, and photorespiratory levels. Examining these datasets in aggregate reveals that ultimately the bulk of the energy flux occurred in the C3 cycle and photorespiration, however, the energy demand from these pathways did not determine the ATP: NADPH demand alone. Instead, a notable contribution was revealed from starch and sucrose synthesis which might counterbalance photorespiratory demand and result in fewer adjustments in mechanisms which balance the ATP deficit.

**Supplementary Information:**

The online version contains supplementary material available at 10.1007/s11120-024-01106-5.

## Introduction

Bioengineering in plants presents an attractive opportunity to exploit their ability to harness light and assimilate CO_2_ through photosynthesis to produce unique specialized metabolites and other bioproducts. Because photosynthesis occurs in the chloroplast and chloroplast transformation results in reproducible expression, this makes chloroplasts a strong target for bioengineering strategies (Strand and Walker 2023). However, to use photosynthetic machinery for bioengineering efforts the need for plants to balance energy harvested from the light reactions with that required by metabolism in order to operate efficiently and avoid damage must be considered (Kramer and Evans 2011; Walker et al. 2020). Energy balancing mechanisms which supply additional ATP and transport reductant from the chloroplast exist to avoid these situations, but little is known regarding how much individual metabolic pathways contribute towards energy demand or the flexibility of such mechanisms in response to dynamic flux through metabolism. Through the perspective of energy balancing, this paper examines how different metabolic pathways contribute to the overall energy demand of the cell with considerations towards how these may impact bioengineering efforts in leaves.

The C3 cycle and photorespiration are thought to require the largest amount of ATP and reductant in photosynthetic tissues (Walker et al. 2020, 2014; Noctor and Foyer 1998). This claim stems in part from the fact that the in vivo activity of rubisco feeds carbon to all downstream metabolism and so is the dominate player in photosynthetic carbon metabolism. The C3 cycle and photorespiration are fed carbon by rubisco following either the carboxylation or oxygenation, respectively, of ribulose 1,5-bisphosphate (RuBP). The C3 cycle initiates when rubisco catalyzes the carboxylation of RuBP to form 2 molecules of 3-phosphoglycerate (3PGA). The 3PGA is converted to 1,3,-bisphosphoglyceric acid and then to glyceraldehyde-3-phosphate of which a portion is ultimately allocated towards starch and sucrose synthesis and the residual regenerates RuBP. Overall, the energetic demand for this process is assumed to be 3 ATP and 2 NADPH per CO_2_ fixed by rubisco. Photorespiration begins with the oxygenation catalyzed by rubisco which produces one molecule of 3PGA and 2-phosphoglycolate (2PG) which through a series of reactions is at least partially converted into 3PGA which enters the C3 cycle. The process occurs across the chloroplast, peroxisome, and mitochondria and ultimately requires 3.5 ATP and 2 NADPH equivalents for each RuBP regenerated (Edwards and Walker 1983). Given the flux of rubisco relative to other enzymes, cellular energetic demands are thought to mostly depend upon the distribution of flux through these pathways which varies based on environmental parameters.

Many key physiological approaches assume that the majority of ATP and NADPH equivalents are consumed by the C3 cycle and photorespiration, with other energy-consuming processes either minor, or constant across measurement conditions. For example, measurements of mesophyll conductance to CO_2_ using chlorophyll fluorescence assume the demand ratios above (Loreto et al. 1992; Gilbert et al. 2011, Warren, 2006). Investigations into the presence of the water-water cycle from rates of oxygen evolution and concurrent gas exchange rely on similar assumptions (Ruuska et al. 2000). These simplifying assumptions are also routinely made to estimate chloroplastic ATP:NADPH demand ratios as discussed further below (Walker et al. 2014). While these simplifications can be useful, they only reflect a portion of the total ATP and NADPH demand in the cell.

Although flux from the C3 cycle and photorespiration dominate central metabolism, additional metabolic processes also alter the energetic landscape and include starch and sucrose synthesis, the tricarboxylic acid (TCA) cycle, the oxidative pentose phosphate pathway (OPPP), lipid biosynthesis, and nitrate fixation. During starch and sucrose synthesis, plants convert carbon to sucrose for transport and starch for storage of carbohydrates. These processes require both ATP and UTP and take place across the chloroplast and cytosol (McClain and Sharkey 2019). The TCA cycle provides precursors for respiration, amino acid biosynthesis, and nitrogen metabolism and ultimately produces some ATP and NADH in the mitochondria (Zhang and Fernie 2018). The OPPP produces NADPH and pentose phosphates. There are two currently proposed OPPP pathways occurring in either the cytosol or the chloroplast, however, the exact location remains an area of active discussion (Xu et al. 2022, 2023a; Wieloch et al. 2023). Lipid biosynthesis is an energetically expensive processes occurring in the chloroplast. Nitrate assimilation requires reducing equivalents and occurs both in the cytosol and in the chloroplast.

It can be useful to evaluate ATP and NADPH flux through individual pathways, however, the ratio of ATP:NADPH demand across many pathways collectively is often more informative from an energy balancing perspective where both nucleoside triphosphates and reductant are referred to in terms of ATP and NADPH. This is partly because the pool sizes of chloroplastic ATP and NADPH are relatively small compared to the high flux being produced from the light reactions and furthermore the light reactions produce ATP and NADPH in a constrained stoichiometry of ~ 1.3 through linear electron flow (LEF) (Hangarter and Good 1982b). The stoichiometry of ATP:NADPH production from the light reactions is constrained due to the coupling of proton and electron transfer. The ~ 1.3 ATP:NADPH production from LEF falls short of the ATP:NADPH demand from the largest ATP:NADPH consuming pathways, the C3 cycle and photorespiration which demand 1.5 and 1.75, respectively (Noctor and Foyer 1998). The discrepancy between supply and demand may lead to an ATP deficit (where ATP is insufficient relative to NADPH) if the chloroplast does not activate alternative ATP generating pathways in addition to LEF (Smith et al. 2023; Strand and Walker 2023). An ATP deficit may lead to metabolic bottlenecks, lower assimilation, and ultimately yields. As outlined above, the ATP:NADPH demand of the cell is often primarily determined by the proportion of flux through either the C3 cycle or photorespiration. However, the question arises as to how additional energy consuming metabolic pathways contribute to the ATP:NADPH demand and ultimately the ATP deficit.

Plants employ various energy balancing strategies to meet fluctuating cellular energetic demand. Within the chloroplast, alternative ATP generating processes are thought to play a large role in balancing an ATP deficit. These alternative pathways are cyclic electron flow (CEF), the malate valve, and the water-water cycle [reviewed in (Allen, 2003a, Scheibe, 2004, Asada, 1999, Kramer and Evans 2011)]. Both CEF and the water-water cycle occur in the chloroplast whereas, the malate valve shuttles reductant between the chloroplast, peroxisome, and mitochondria. Given that metabolism and energy balancing occur across several organelles this then poses the question of how the distribution of ATP and NADPH from supplying processes is coordinated with consumption throughout the cell.

One approach for determining how each individual metabolic pathway contributes to the ATP:NADPH demand is to leverage metabolic flux networks. Isotopically non-stationary metabolic flux analysis (INST-MFA) is an increasingly used technique in plants for quantifying metabolic flux through central metabolism (Jazmin et al. 2014). Typically, the scope of these studies focuses on carbon flux, however, they can be used to resolve cellular energy flux by coupling each reaction to its associated ATP and NADPH demand. By evaluating the energy demand from different pathways, we can gain insight into how these pathways specifically contribute to ATP and NADPH demand. In this study, we used flux values from 8 different INST-MFA experiments which consist of a variety of species including *Arabidopsis thaliana*, *Nicotiana tabacum*, and *Camelina sativa* across environmental factors such as high/low light, day length, and photorespiratory levels allowing us to characterize the flux of energy across different pathways and compartments (Ma et al. 2017; Fu et al. 2023; Xu et al. 2021, 2022, 2023b). We found that while most of the energy demand did arise from the C3 cycle and photorespiration, the energy demand from these pathways did not account for total cellular demand with notable contributions from starch and sucrose synthesis. Ultimately, we suggest starch and sucrose synthesis may help counterbalance the energy demand from photorespiration to avoid the need for rapid adjustments in alternative ATP generating processes which may limit photosynthetic efficiency or cause photodamage.

## Materials and methods

We compiled data from 8 different ^13^C labeled INST-MFA data sets to use in our analysis. Within the INST-MFA data sets, individual reactions were grouped into their cellular compartment and respective pathways (Ma et al. 2017; Fu et al. 2023; Xu et al. 2021, 2022). The pathways we included in our analysis were the C3 cycle, photorespiration, starch and sucrose synthesis, the TCA cycle, and the OPPP as almost every data set had reported flux through these pathways with the exception of the OPPP being absent in *Arabidopsis thaliana* data sets (ATP and NADPH demands for each flux map available as supplementary material). Some other pathways which were excluded from our analysis include lipid biosynthesis and nitrate assimilation due to not having reported metabolite flux into those pathways. By categorizing the carbon flux from the individual reactions described below, we could calculate the energy demand for the various pathways and compartments. For the C3 cycle, we used the flux from 3PGA to triose phosphates (− 1 ATP, − 1 NADPH per turnover) and ribulose 5-phosphate (Ru5P) to RuBP (− 1 ATP per turnover). For photorespiration, we used the carbon flux for glycine to serine (+ 1 NADH, − 1 ATP, − 2 Fd assuming the NH_4_^+^ recapture per turnover), hydroxypyruvate to glycerate (− 1 NADH per turnover), and glycolate to 3PGA (− 1 ATP per turnover) where the stoichiometry of 2 glycine to 1 serine is already accounted for within the flux solution as part of the model structure. For the OPPP, we used carbon flux for glucose-6-phosphate to 6-phosphogluconolactone (6PG, + 1 NADPH per turnover) and 6PG to Ru5P (+ 1 NADPH per turnover). For starch and sucrose synthesis, we used carbon flux for glucose-1-phosphate to ADP glucose (− 1 ATP per turnover) and glucose-1-phosphate to UDP-glucose (− 1 UTP per turnover). We additionally considered flux from starch and sucrose cycling by using carbon flux from fructose to fructose-6-phosphate (− 1 ATP per turnover) and glucose to glucose-6-phosphate (− 1 ATP per turnover). For the TCA cycle we used phosphoenolpyruvate to pyruvate (+ 1 ATP per turnover), isocitrate to alpha-ketoglutarate (AKG, + 1 NADH per turnover), AKG to succinate (+ 1 NADH, + 1 GTP per turnover), succinate to fumarate (+ 1 FADH_2_ per turnover), malate to oxaloacetate (+ 1 NADH per turnover), AKG to glutamate (− 1 NADH per turnover), and pyruvate to acetyl-CoA (+ 1 NADH per turnover). Despite the fact that these reactions use a variety of energy carriers as outlined above, for simplicity sake we will refer to reductant in terms of NADPH equivalents and nucleoside triphosphates in terms of ATP.

The percentage of ATP needed to be supplied by alternative ATP generating processes was calculated by subtracting the assumed 1.3 ATP:NADPH demand supplied by LEF from the ATP:NADPH demand from metabolism for each data set. This value was then divided again by the 1.3 value provided from the light reactions and multiplied by 100.

1 $${\text{Percentage of ATP needed from alternative electron processes}} = \left( {\frac{{{\text{ATP}}:{\text{NADPH demand}} - 1.3}}{1.3}} \right) \times 100$$

Data handling, statistical analysis, and graphing were performed in R using dplyr and ggplot2 packages.

## Results

### Most leaf energy demand in the chloroplast comes from the C3 cycle and photorespiration

**Fig. 1:**
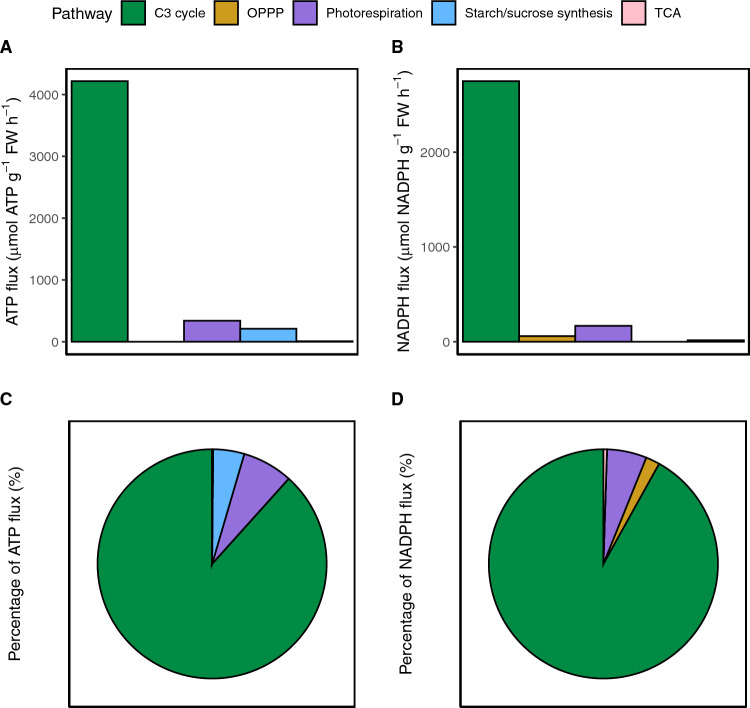
The relative flux of ATP and NADPH through individual metabolic pathways. Relative flux of (**A**) ATP and (**B**) NADPH through individual pathways were determined by finding the absolute value of the total energetic flux from each pathway then averaging across all data sets. Data sets which manipulated levels of photorespiration using altered O_2_ concentrations were not included. The fluxes were then expressed in the percentage of the total (**C**) ATP and (**D**) NADPH flux

As described above, the largest energy-consuming pathways in photosynthetic tissues are thought to be the C3 cycle and photorespiration, however, the plant cell uses ATP and NADPH in a multitude of other pathways. To determine how much each pathway contributes to the total flux, we examined the flux from individual pathways by finding the absolute value of the energetic flux from each pathway and averaging this across all the flux data sets excluding those which manipulated levels of photorespiration using altered O_2_ concentrations. ATP and NADPH energy use was the highest in the C3 cycle, with it requiring 4.2 mmol ATP g^−1^ FW h^−1^ and 2.7 mmol NADPH g^−1^ FW h^−1^ which was ~ 88% of ATP and ~ 91% of NADPH total flux (Fig. 1). Next largest was photorespiration with 0.34 mmol ATP g^−1^ FW h^−1^ and 0.17 mmol NADPH g^−1^ FW h^−1^ or about ~ 7% total of ATP and ~ 6% NADPH flux. Interestingly, the OPPP produced 59 µmol NADPH g^−1^ FW h^−1^ or 2% of total NADPH flux. Starch and sucrose synthesis required 0.21 mmol ATP g^−1^ FW h^−1^ or about 4% of total ATP flux. Finally, the TCA cycle produced 7 µmol ATP g^−1^ FW h^−1^ and 15 µmol NADPH g^−1^ FW h^−1^ which was less than 1% of total ATP and NADPH flux. This flux includes the flux of carbon skeletons that are modeled to leave the TCA cycle for amino acid synthesis. These results highlight that, while C3 and photorespiration do dominate metabolism, small contributions from other pathways may have important roles in energy balancing. For example, if CEF must operate at 13% of LEF to supply the C3 cycle, then starch and sucrose synthesis consuming 4% of the total ATP demand may substantially impact the system (Strand et al. 2017).

**Fig. 2:**
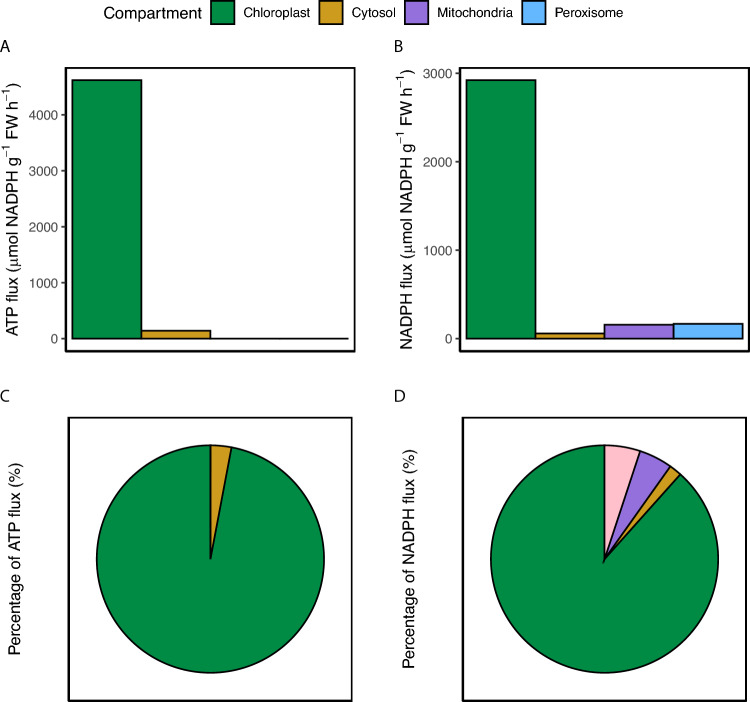
The relative flux of ATP and NADPH through individual compartments. Relative flux of (**A**) ATP and (**B**) NADPH through individual compartments were determined by finding the absolute value of the total energetic flux from each compartment then averaging across all data sets. Data sets which manipulated levels of photorespiration using altered O_2_ concentrations were not included. The fluxes were then expressed in the percentage of the total (**C**) ATP and (**D**) NADPH flux

Metabolism acts across all cellular compartments, requiring the plant to coordinate and transport ATP and NADPH. This need for this coordination invites the question of how the distribution of ATP and NADPH consumption is coordinated throughout the cell and how are these demands met. To examine the energy flux in individual compartments, we took the absolute value of the energetic flux from each pathway and averaged this across all the flux data sets again excluding those which manipulated levels of photorespiration through altered oxygen concentrations. We found ATP and NADPH flux was highest in the chloroplast with it requiring 4.6 mmol ATP g^−1^ FW h^−1^ and 2.9 µmol NADPH g^−1^ FW h^−1^ which was 97% of total ATP flux and 88% of total NADPH flux (Fig. 2). Within the peroxisome, 0.17 mmol NADPH g^−1^ FW h^−1^ was required which was 5% of NADPH flux. ATP and NADPH flux in the cytosol was 0.14 mmol ATP g^−1^ FW h^−1^ consumed and 59 µmol NADPH g^−1^ FW h^−1^ produced which was 3% of ATP flux and 2% of NADPH flux. 0.16 mmol NADPH g^−1^ FW h^−1^ was produced in the mitochondria which was 5% of NADPH flux. This not only highlights the large amounts of ATP and NADPH required in the chloroplast but also the amount of NADPH required in the peroxisome, and it’s role as an energetically important organelle.

### Cellular ATP:NADPH demand and the percentage of ATP supplied by alternative ATP generating processes varied amongst species and conditions

**Fig. 3:**
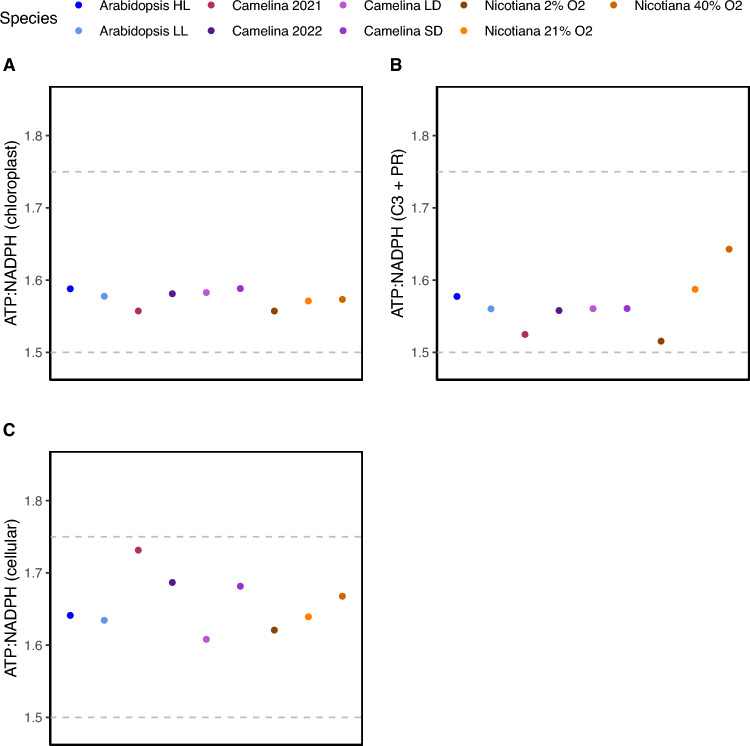
The dynamicity of the ATP:NADPH demand across different conditions and species. Shown are the ATP:NADPH demand for the different data sets at the scale of **A** the chloroplast, **B** from solely the C3 cycle and photorespiration (PR), and **C** cellular. ATP:NADPH demand were calculated by totaling the ATP and NADPH demand separately from the carbon flux then taking the ratio of ATP:NADPH. The dashed gray lines represent the energy demand for the C3 cycle (lower) and photorespiration (upper)

The ATP deficit in the plant varies based on the proportion of flux through downstream metabolism which changes based on environmental conditions, however, the range of this variation is under-explored. We first examined the range of the ATP deficit by examining the ATP:NADPH demand in the chloroplast because it contains the majority of metabolic flux. ATP:NADPH demand was most similar between species and conditions in the chloroplast where demand ranged from 1.56 to 1.59 (Fig. 3A). Since the majority of chloroplastic flux is from the C3 cycle and photorespiration, the demand in the chloroplast should be almost identical to that from the C3 cycle and photorespiration. Indeed, these ratios were almost identical to the ATP:NADPH demand solely from photorespiration and the C3 cycle, the exception being the 2%, 21% and 40% O_2_ *Nicotiana tabacum* data sets likely from the inclusion of mitochondrial and peroxisomal reactions which introduced additional demand related to photorespiration (Fig. 3B). This suggests that the ATP deficit experienced specifically within in the chloroplast is relatively similar between species and conditions such as gas concentrations, growth day length, and light intensities.

Given that the largest energy demand was found to be in the chloroplast with 97% of total ATP flux and 88% of total NADPH flux within the whole cell, it seemed likely that the cellular ATP: NADPH demand would also be relatively similar to the chloroplast between species and conditions (Fig. 2). However, while the extent of ATP deficit varied little in the chloroplast, the total cellular ATP: NADPH demand was more variable and higher overall. More specifically, the demand among the different conditions ranged from 1.61 to 1.73 (Fig. 3C). The larger variability among the environmental conditions in the species in the whole cell compared to the chloroplast alone suggests the potential for a larger role of other metabolism in determining the total ATP: NADPH demand rather than being solely determined by flux through the C3 cycle and photorespiration.

Ultimately, all the conditions above support the presence of an ATP deficit because they are all greater than the ~ 1.3 ATP: NADPH supplied by LEF. The discrepancy in ATP: NADPH between supply and demand must therefore be met by alternative ATP generating processes such as CEF, the malate valve, and the water-water cycle. We calculated the percentage of the ATP:NADPH which must be met by alternative ATP generating processes both in the whole cell as well as the chloroplast specifically for each data set. We found ATP demand which must be supplied by alternative ATP generating processes was between 24 and 33% in the whole cell from the metabolism measured in the component MFA analyses. In the chloroplast specifically the ATP demand which must be supplied by alternative processes was reduced to 19–22% (Table 1). The discrepancy between the percentage of ATP demand which must be met by alternative processes in the whole cell and the chloroplast raises questions as to how the plant employs different energy balancing processes and their flexibility under dynamic conditions.

**Table 1:** ATP needed to be supplied by alternative ATP generating processes

Dataset	ATP needed to be supplied by alternative electron processes in the whole cell (%)	ATP needed to be supplied by alternative electron processes in the chloroplast (%)
Arabidopsis HL	26	22
Arabidopsis LL	26	21
Camelina LD	33	19
Camelina SD	29	21
Camelina 2021	23	21
Camelina 2022	29	22
Nicotiana 21% O_2_	24	19
Nicotiana 2% O_2_	26	20
Nicotiana 40% O_2_	28	21

Shown is the percentage ATP needed to be supplied by alternative electron processes in both the whole cell and the chloroplast for each data set. Details of these calculations are found in the text

### ATP and NADPH demand are reasonably estimated by rates of rubisco carboxylation and oxygenation

**Fig. 4:**
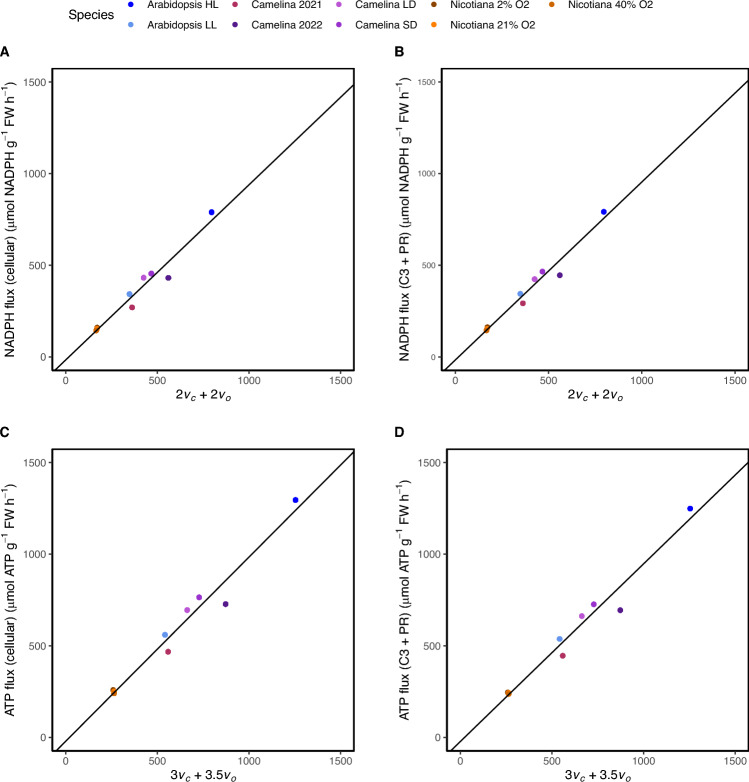
Comparison between the values calculated for ATP and NADPH fluxes. First, shown are the comparisons between the estimated NADPH flux as calculated by adding the products of vc and vo multiplied by the NADPH demand from the C3 cycle and PR, respectively, with the NADPH flux through **A** the cell (r^2^ = 0.94, p < 0.05, linear regression model) **B** through solely the C3 cycle and photorespiration (PR) (r^2^ = 0.96, p < 0.05, linear regression model). Then, then same comparisons are made for ATP flux through **C** the cell) (r^2^ = 0.96, p < 0.05, linear regression model) and **D** and solely the C3 cycle and PR) (r^2^ = 0.96, p < 0.05, linear regression model). The solid black lines represent the results of the results of the linear regression models

Due to difficulties associated with measuring ATP and NADPH demand related to their fast turnover times, one method of assessing the ATP and NADPH demand has been approximating the levels from the rate of rubisco carboxylation (*v*_c_) and the rate of rubisco oxygenation (*v*_o_) estimated from leaf gas exchange (Walker et al. 2020; Hangarter and Good 1982a, Sharkey, 1988). This demand is calculated by adding the products of *v*_c_ and *v*_o_ multiplied by the requirements for the C3 cycle (3 ATP and 2 NADPH) and photorespiration (3.5 ATP and 2 NADPH). Since energy demand estimates from *v*_c_ and *v*_o_ rely solely on the contributions from the C3 cycle and photorespiration, we compared demand estimated from *v*_c_ and *v*_o_ to not only flux data demand from the entire cell but as well to as solely from the C3 cycle and photorespiration. Overall, both total NADPH and ATP demand correlated strongly with demand calculated from *v*_c_ and *v*_o_ (Fig. 4). Total ATP demand correlated the identically with both the entire cell and from the C3 cycle and photorespiration (Fig. 4C-D). NADPH demand correlated slightly less in the entire cell when compared to from the C3 cycle and photorespiration (Fig. 4A-B). Previously ATP:NADPH has been estimated from multiplying *v*_c_ and *v*_o_ by the respective demand outlined above for the C3 cycle and photorespiration (Walker et al. 2020). The data suggests the method reasonably estimates energetic demands calculated from flux networks. However, there are slight imperfections as the relationships are not perfectly linear with the least linear being the cellular NADPH demand (Fig. 4A).

### In addition to the C3 cycle and photorespiration, starch and sucrose contribute notably to cellular ATP:NADPH demand

**Fig. 5:**
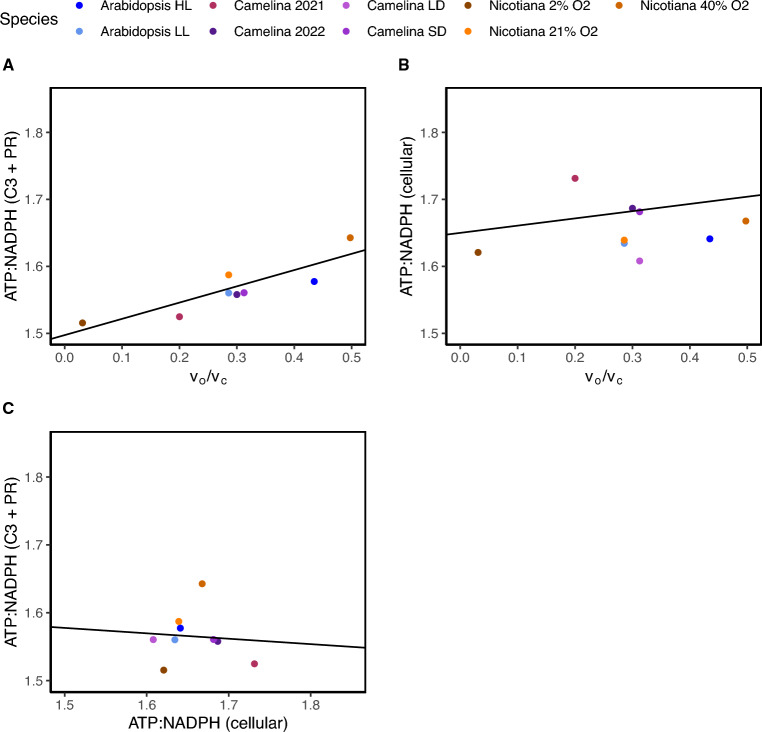
The contribution of the C3 cycle and photorespiration in determining cellular ATP:NADPH demand. First, **A** the relationship between the ATP:NADPH demand from the C3 cycle and photorespiration and *v*_o_/*v*_c_ (r^2^ = 0.76, p < 0.05, linear regression model). Then, **B** the relationship between cellular ATP:NADPH and vo/vc (r^2^ = 0.0.007, p > 0.05, linear regression model) and finally between **C** the ATP:NADPH demand from the C3 cycle and photorespiration and cellular ATP:NADPH demand (r^2^ = 0.003, p > 0.05, linear regression model). The solid black lines represent the results of the results of the linear regression models

Since a majority of energetic demand comes from the C3 cycle and photorespiration, we hypothesized that most of the variability in the cellular ATP:NADPH demand between the individual data sets may be due to differences in the flux through these pathways. If this were the case, then the ATP:NADPH demand should be proportional to *v*_o_/*v*_c_. ATP:NADPH demand from just the C3 cycle and photorespiration correlated with rates of *v*_o_/*v*_c_ more weakly than expected (but still significant) likely due to the slight imperfections in estimating ATP and NADPH demand described above (Fig. 5A, r^2^ = 0.76, p < 0.05, linear regression model). Interestingly, cellular ATP:NADPH for core metabolism and *v*_o_/*v*_c_ were only weakly and insignificantly correlated (Fig. 5B, r^2^ = 0.007, p > 0.05). We then examined whether ATP:NADPH from the C3 cycle and photorespiration correlated with cellular demand and found an almost non-existent correlation (Fig. 5C, r^2^ = 0.003, p > 0.05, linear regression model). This suggests that while a majority of energy demand does come from the C3 cycle and photorespiration, differences in flux in these pathways do not entirely explain the increase or variation in ATP:NADPH demand.

**Fig. 6:**
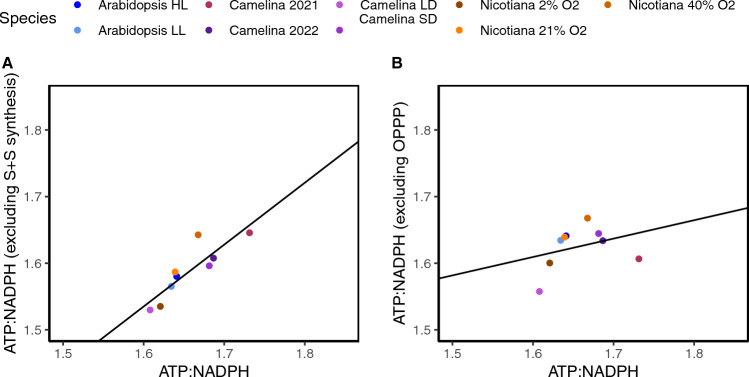
The removal of metabolic pathways to evaluate their contribution towards the cellular ATP:NADPH. First, removal of **A** starch and sucrose (S + S) synthesis (r^2^ = 0.76, p < 0.05, linear regression model) and **B** the OPPP (r^2^ = 0.11, p > 0.05, linear regression model) to evaluate their contribution in determining cellular ATP:NADPH demand. The solid black lines represent the results of the results of the linear regression models

**Fig. 7:**
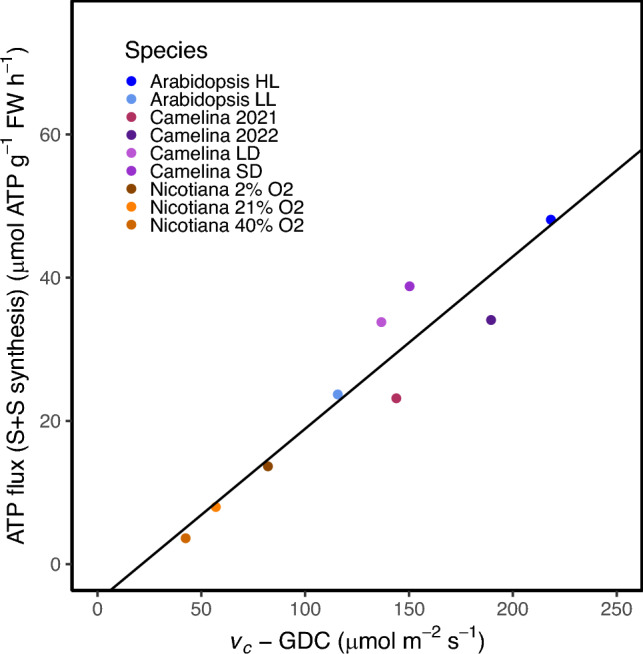
The relationship between ATP flux through starch and sucrose synthesis and the net carbon fixed. Shown is the relationship between the ATP flux through starch and sucrose synthesis (S+S) and the net carbon fixed as calculated by the rate of rubisco carboxylation (*v*_c_) minus the flux through the glycine decarboxylase complex (GDC). ATP flux through starch and sucrose synthesis was found by taking the absolute value of the sum of the ATP flux through each ATP consuming reaction in starch and sucrose synthesis for every data set. The solid black line displays the results of the linear regression model (r^2^ = 0.90)

Since the proportion of flux through the C3 cycle and photorespiration did not appear to determine cellular ATP:NADPH demand, we hypothesized a different metabolic pathway must also play a prominent role. Based on the ATP and NADPH flux values, it is likely that the increase in demand was arising from either the NADPH produced by the OPPP, or the ATP required from starch and sucrose synthesis (Fig. 1). To test this, we alternatively subtracted energy consumption from either the combined starch and sucrose synthesis or the energy produced from the OPPP to observe which would increase the correlation with cellular ATP:NADPH. When starch and sucrose biosynthesis was removed the relationship improved (Fig. 6A, r^2^ = 0.76, p < 0.05, linear regression model). However, when the OPPP was removed the relationship only slightly improved (Fig. 6B, r^2^ = 0.11, p > 0.05, linear regression model). This suggests that aside from the C3 cycle and photorespiration, starch and sucrose synthesis has the largest effect on cellular ATP:NADPH demand with a smaller role for the OPPP. To determine how the energy demand from starch synthesis scaled with net carbon assimilation, we investigated the relationship between the ATP required from starch and sucrose biosynthesis and *v*_c_ with the flux through the glycine decarboxylase complex subtracted to account for carbon loss from photorespiration. ATP consumption from starch and synthesis did scale relatively well with this net carbon assimilation displaying how ATP demand from starch and sucrose synthesis scales with net assimilation (Fig.7).

## Discussion

### Starch and sucrose synthesis may help counterbalance photorespiration to maintain cellular energy demand

These findings support the hypothesis that starch and sucrose synthesis counterbalance changes in cellular ATP: NADPH demand when accompanied by changes in net assimilation. Typically, cellular ATP: NADPH demand is thought to be determined mainly from the distribution of flux through photorespiration and the C3 cycle due to the large flux of these pathways (Fig. 1) (Walker et al. 2020; Smith et al. 2023). If this were true, cellular ATP: NADPH demand would decrease or increase according to levels of photorespiration due to the extra ATP consumption from this pathway. However, in this study we show that the ATP: NADPH demand from the C3 cycle and photorespiration did not entirely explain cellular demand (Fig. 4). Instead, starch and sucrose synthesis also substantially contributed to cellular ATP:NADPH demand (Fig. 5A). We propose this is because as photorespiration is reduced and net assimilation increases, there is more flux into carbon assimilation ultimately which also increases starch and sucrose synthesis (Fig. 7, (Fu et al. 2023, Sharkey et al. 1985). Therefore, since starch and sucrose synthesis and photorespiration both increase the cellular ATP required as they inversely change perhaps this helps maintain a more similar ATP:NADPH demand.

Perhaps the inverse relationship between levels of photorespiration and starch and sucrose synthesis begins to explain why decreasing either result in a similar effect on the light reactions. First, both triose phosphate utilization (TPU) and photorespiration result in a substrate limitation on ATP synthase. TPU, related to starch and sucrose synthesis, serves as a biochemical limitation of photosynthesis in C3 plants when plants are photosynthesizing rapidly and the turnover of triose phosphates into end products causes a decrease in available phosphate limiting the rate of photosynthesis via ATP synthase (McClain and Sharkey 2023; Kiirats et al. 2009). Similarly, when photorespiration is decreased it also results in a decrease in ATP synthase conductivity likely due to a decrease in ATP consumption (Smith et al. 2023). Additionally, when either of these metabolic pathways are disrupted, mechanisms that supply additional ATP, such as CEF, appear to be affected. For example, CEF increases when the flow of triose phosphates into sucrose is altered via a mutation in the C3 cycle enzyme fructose 1,6-bisphosphatase (Livingston et al. 2010a). Likewise, there is an increase in CEF when the flow of the photorespiratory pathway is interrupted via a peroxisomal hydroxypyruvate reductase (*hpr1*) knockout (Li et al. 2019). This increase in CEF causes increased acidification of the lumen which also induces photo-protective NPQ, but it also increases the ATP produced relative to LEF. Both of these instances occur due to an increase in peroxide as potentially a signal that metabolism has been disrupted. Overall, the similar impacts on both the light reactions and CEF when either starch and sucrose synthesis or photorespiration are impaired further highlight the potential role of starch and sucrose synthesis aiding in counterbalancing photorespiration to maintain similar cellular ATP:NADPH demand.

### Contributions from starch and sucrose synthesis to ATP:NADPH demand could decrease the need for rapid adjustments from alternative ATP generating processes in dynamic environments.

The extent of the variation of ATP:NADPH demand under different conditions and species has not been explored, however, here we show that the chloroplast ATP:NADPH demand varied less than cellular ATP:NADPH demand between various species and conditions. The chloroplast ATP:NADPH demand was almost identical to the ATP:NADPH demand solely the C3 cycle and photorespiration with the exception of the *Nicotiana tabacum* data sets likely because these data sets directly manipulate levels of photorespiration (Fig. 3). This suggests that the ATP deficit specifically within in the chloroplast is relatively similar between species and conditions such as day length and light intensities. However, we found while the extent of ATP deficit varied little in the chloroplast, the total cellular ATP:NADPH demand was more variable with higher overall demand (Fig. 3C). The variability appears to be due to differences in the flux in starch and sucrose biosynthesis between the species and conditions.

The counterbalance between photorespiration and starch and sucrose synthesis would be beneficial because it would decrease the need to dynamically adjust alternative ATP generating processes as cellular ATP:NADPH demand varies. As shown here, the percentage of alternative processes needed to supply ATP can be quite substantial and ranged between 24–33% in the whole cell (Table 1). The increased variability in the whole cell ATP:NADPH demand observed under different environmental conditions and species suggests cellular demand is likely rapidly changing according to the environment (Fig. 3C). The proposed counterbalance between starch and sucrose synthesis and photorespiration could be beneficial to avoid potentially rather large adjustments in alternative processes which could be experienced under dynamic environments. For example, if the plant were to experience an increase in ATP:NADPH demand and then accordingly increase CEF this would result in an increase in proton motive force and ultimately the downregulation of the light reactions and photosynthesis (Livingston et al. 2010b; Alric and Johnson 2017, Allen, 2003). This perhaps begins to resolve why altering flux through the C3 cycle or photorespiration does not always appear to activate CEF as expected from changes in energetic demands or under sub-saturating light intensities (Smith et al. 2023; Walker et al. 2014).

### C3 cycle and photorespiration within the chloroplast represented the majority of energy demand

Ultimately, the flux from starch and sucrose synthesis is still small proportionally to the large flux from the C3 cycle and photorespiration. The C3 cycle represented most of the flux with ~ 88% of ATP and ~ 91% of reductant and photorespiration accounted for only ~ 7% of ATP and ~ 6% of reductant. This proportional energy demand for photorespiration is much lower than previous estimates, however, this is because other calculations partition the energy required to regenerate the 3PGA recycled from photorespiration back into Ribulose-5-phosphate, whereas ours account for this energy as part of the C3 cycle (Walker et al. 2016; Edwards and Walker 1983). We chose to assign energy accounting strictly according to the reactions where it occurred to better align with the flux solutions, but recognize that a full life cycle analysis of photorespiration using just the *v*_o_ and *v*_c_ values with these datasets would result in similar numbers as past studies. In contrast, starch and sucrose synthesis accounted for about 4% of ATP consumption (Fig. 1). It should be noted that this value would increase if futile cycling of sucrose was accounted for, but this cycling was not resolved in the flux datasets we analyzed. These calculations highlight that while other metabolic pathways may have more energetic significance than expected, the C3 cycle and photorespiration still dominate in terms of absolute flux.

Given the large amounts of ATP and NADPH required by the C3 cycle and photorespiration as calculated here, bioengineering efforts focused on decreasing the rate of photorespiration may need to consider maintaining native energy demands to not perturb the energetic balance between supply and demand. Recently, bioengineering efforts have focused on decreasing the CO_2_ and NH_4_^+^ released from photorespiration to improve photosynthetic efficiency (South et al. 2019; Kebeish et al. 2007; Basler et al. 2016; Peterhansel et al. 2013b). However, strategies for reengineering photorespiration, commonly referred to as photorespiratory bypasses, also impose novel energetic requirements which may not be met by intrinsic cellular energetic flexibility. These bypasses alter the total ATP and NADPH required by photorespiration and as a result the ATP:NADPH ratio increases or decreases depending on the engineered pathway (Peterhansel et al. 2013a). An increase or decrease in the ATP:NADPH ratio may negatively impact the light reactions or the regulation of alternative processes, highlighting the need to resolve the fundamental bioenergetics linked to photorespiration. More specifically, when the ATP:NADPH demand decreases due to reduced photorespiration this causes a decrease in electron transfer and may eventually limit crop yields (Smith et al. 2023).

While this study deepens our overall understanding of how many different metabolic pathways contribute to the cellular ATP:NADPH demand, the exclusion of nitrate assimilation and lipid biosynthesis presents an inherent limitation of this analysis. Since nitrate assimilation and lipid biosynthesis were not measured in the available studies, and their variation across species and conditions is not well quantified, we did not include them in this particular meta-analysis but it is still important to contextualize how these data might have impacted the model. Estimates for the reductant demand of nitrate assimilation suggest the pathway requires ~ 2.5–23% of total NADPH demand (Walker et al. 2020). Given the large amount of reductant potentially required, the inclusion of nitrogen assimilation into our model would likely represent a large proportion of total reductant flux. Previous studies have already highlighted the potential influence of nitrogen assimilation on energy balancing and argue that the process should be considered in the ATP balance during C3 photosynthesis (Noctor and Foyer 1998). With respect to lipid biosynthesis, the estimated NADPH demand to maintain turnover rate of fatty acid is approximately 0.5–2% of total NADPH demand which is most likely insufficient to affect calculations of total leaf energy balancing (Walker et al. 2020).

### The complex energetic interplay between the peroxisome and the mitochondria

In this analysis we explored how metabolism acts across several organelles as well as in the exterior cytosol. While the chloroplast required the most ATP and NADPH, there was a larger contribution from the peroxisome than we anticipated. More specifically, the chloroplast required approximately ~ 97% of the ATP and ~ 88% of the NADPH compared to the other cellular compartments (Fig. 2). Additionally, the peroxisome required approximately ~ 5% of the reducing power.

It is often assumed that the conversion of glycine to serine during photorespiration in the mitochondria produces reductant which offsets the demand in the peroxisome for glycerate synthesis (Raghavendra et al. 1998). These processes are then accordingly linked in corresponding metabolic flux models and then resulted here in the 5% of the reductant produced in the mitochondria matching the 5% reductant required in the peroxisome. However, there is evidence glycine can be exported from photorespiration, which would reduce the reductant ultimately available to the peroxisome by decreasing the stoichiometry of glycine decarboxylation (Fu et al. 2023; Harley and Sharkey 1991; Busch et al. 2018). Albeit this glycine export likely occurs at low rates and would therefore not largely reduce the reductant available. Additionally, the reductant produced in the conversion of glycine to serine could be used in the mitochondrial electron transport chain, which would lead to a decrease in reductant supply to the peroxisome (Shameer et al. 2019). If this were true, it is possible some of the reductant not provided from the mitochondria could be provided to the peroxisome from the 2% of NADPH produced by the OPPP as observed here.

## Conclusions

Overall, this work highlights how the energetic landscape is diverse and complicated. While the C3 cycle and photorespiration represented most of the energetic flux, additional metabolic pathways, most notably starch and sucrose synthesis, drove variation in energy demands between species and environmental conditions. Additionally, the contribution of starch and sucrose synthesis in ATP:NADPH demand proposed an important mechanism for homeostasis in the complicated energetic landscape. Ultimately, by deepening our understanding of the interactions within the energetic landscape we can more accurately inform and address challenges in metabolic engineering to increase both crop yields and desired products.

### Supplementary Information

Below is the link to the electronic supplementary material.

Supplementary file1 (CSV 15 KB)

## Data Availability

We have indicated that the data is avialable in the supplmentary material in the methods section. “ATP and NADPH demands for each flux map available as supplementary material”.
